# Microencapsulation of *Mitragyna* leaf extracts to be used as a bioactive compound source to enhance *in vitro* fermentation characteristics and microbial dynamics

**DOI:** 10.5713/ab.23.0200

**Published:** 2023-11-01

**Authors:** Maharach Matra, Srisan Phupaboon, Pajaree Totakul, Ronnachai Prommachart, Assar Ali Shah, Ali Mujtaba Shah, Metha Wanapat

**Affiliations:** 1 Tropical Feed Resources Research and Development Center, Department of Animal Science, Faculty of Agriculture, Khon Kaen University, Khon Kaen 40002, Thailand; 2 Division of Animal Science, Faculty of Agricultural Technology, Rajamangala University of Technology Thanyaburi, Thanyaburi, Pathum Thani 12130, Thailand; 3 Department of Animal Science, Faculty of Agriculture and Natural Resources, Rajamangala University of Technology, Tawan-Ok 20110, Thailand; 4 Department of Livestock Production, Shaheed Benazir Bhutto University of Veterinary and Animal Science, Sakrand 67210, Sindh, Pakistan

**Keywords:** Bioactive Compounds, Microencapsulation, *Mitragyna Speciosa* Korth, Rumen Fermentation, Ruminants

## Abstract

**Objective:**

*Mitragyna speciosa* Korth is traditionally used in Thailand. They have a high level of antioxidant capacities and bioactive compounds, the potential to modulate rumen fermentation and decrease methane production. The aim of the study was to investigate the different levels of microencapsulated-*Mitragyna* leaves extracts (MMLE) supplementation on nutrient degradability, rumen ecology, microbial dynamics, and methane production in an *in vitro* study.

**Methods:**

A completely randomized design was used to assign the experimental treatments, MMLE was supplemented at 0%, 4%, 6%, and 8% of the total dry matter (DM) substrate.

**Results:**

The addition of MMLE significantly increased *in vitro* dry matter degradability both at 12, 24, and 48 h, while ammonia-nitrogen (NH_3_-N) concentration was improved with MMLE supplementation. The MMLE had the greatest propionate and total volatile fatty acid production when added with 6% of total DM substrate, while decreased the methane production (12, 24, and 48 h). Furthermore, the microbial population of cellulolytic bacteria and *Butyrivibrio fibrisolvens* were increased, whilst *Methanobacteriales* was decreased with MMLE feeding.

**Conclusion:**

The results indicated that MMLE could be a potential alternative plant-based bioactive compound supplement to be used as ruminant feed additives.

## INTRODUCTION

Tropical plants are rich in bioactive compounds (BC) namely phenolic, flavonoid compounds, and antioxidant capacities [1,2], which may have anti-microbial effects, especially in methanogen and protozoal populations, and which improve the characteristics of rumen fermentation [3,4]. The BC have been demonstrated to influence product quality and health condition that play a vital role in animal nutrition [5].

One of the alternative sources of plants containing BC is *Mitragyna speciosa* Korth, a tropical plant in Southeast Asia including Myanmar, Malaysia, and Thailand [6]. *M. speciosa* is popularly known as Kratom in Thailand. They are traditionally used to treat tiredness, opioid addiction, and relieve pain [7]. The leaves of *M. speciosa* have shown the presence of BC such as flavonoids, alkaloids, glycoside, and triterpenoids [8]. This plant has been demonstrated to have several pharmacological properties such as antibacterial, anti-inflammatory, and antioxidant [9]. Accordingly, Phesatcha et al [10] reported that the *Mitragyna* leaf powder supplementation as a BC can improve rumen fermentation, whilst decrease rumen protozoa, and methane production. Chanjula et al [11] revealed that *Mitragyna* leaf powder enhanced rumen ecology by increasing nutrient digestibility, volatile fatty acid (VFA, propionic acid profile), and reducing methane production in goats.

Microencapsulation is an emerging technology that is commonly used nowadays in animal nutrition for the preparation of stable products (vitamins, minerals, fatty acids, as well as BC) [12]. This technique can act as a physical barrier to protect pharmaceuticals from harsh external environment, which increases the stability of the substance [13]. Among microencapsulation procedures, spray-drying is a practical approach that could produce a constant microcapsule [14]. However, no previous research has evaluated protection of BC by microencapsulation technique of *Mitragyna* leaves as a strategy to enhance their interactions with ruminal fermentation. Therefore, this study aimed at testing the susceptibility of microencapsulated BC from *Mitragyna* leaves to *in vitro* nutrient degradation, rumen ecology, and microbial diversity.

## MATERIALS AND METHODS

### Animal ethics

The collection of rumen fluid from Thai-crossbred dairy cows was permitted by the Institute of Animals for Scientific Purpose Development (IAD), Thailand (number U1-06878-2560).

### Microencapsulated-*Mitragyna* leaves extracts preparation

The plant sources were harvested at Rajamangala University of Technology Srivijaya (MUTSV), Nakhon Si Thammarat, Thailand. Fresh *Mitragyna* leaf was dried at 60°C. The dried *Mitragyna* was ground through a sieve opening of 1 mm (Cyclotech Mill, Tecator, Hoganas, Sweden). The powder was mixed with water and heated in a Microwave to 60°C, and after 35 minutes the particulates filtered out. The liquids were combined with tween 80 and chitosan [15], and they were spray-dried microencapsulated-*Mitragyna* leaves extracts (MMLE) by using Bǚchi B-191 Mini Spray Dryer [16]. The surface morphology of MMLE was observed using a field-emission scanning electron microscope (FE-SEM; model: Mira, Tescan Co., Brno, Czech Republic) according to Ko et al [17]. MMLE were chemically analyzed for dry matter (DM; number 967.03), ash (number 942.05), and crude protein (CP; number 984.13) following the methods of AOAC [18], as shown in number 973.18. Fiber fractions (neutral-detergent fiber [NDF] and acid-detergent fiber [ADF]) were determined using Ankom A200i Fibre Analyser (Ankom Technology Co., New York, USA); according to Van Soest et al [19]. MMLE were analyzed for BC especially total phenolic compound (TPC) using Folin–Ciocalteu reagent by absorbance at 765 nm [20] and total flavonoid compound (TFC) following the method of Topçu et al [21], based on colorimetric changes with a 10% aluminum chloride solution read at 415 nm. Moreover, the sample was analyzed the antioxidant capacities including 2,2-diphenyl-1-picrylhydrazyl (DPPH) [22], 2, 2′-azino-bis (3-ethylbenzothiazoline-6-sulfonic acid) (ABTS) [23], and ferric reducing antioxidant power (FRAP) [24], which are additional explained in Phupaboon et al [25].

### Experimental design and treatments

The study was assigned in a completely randomized design (CRD). Total dietary substrates (the ratio of rice straw to concentrate at 60:40) were weighed at 0.5 g into the 60 mL bottles, then the treatments were supplemented with MMLE at 0%, 4%, 6%, and 8% of total DM substrate, respectively.

### Rumen fluid collection and preparation

The rumen fluid donors were four Thai-crossbred dairy cows (body weight, 400±10 kg). The animals consumed total mixed ration twice daily at 7:00 and 16:00 o’clock, and they had unlimited access to mineral block and clean water for at least 14 days following the National Research Council (NRC) [26] requirement for dairy cows. Samples of the rumen fluid were taken using a tube connected with a vacuum pump set through the mouth to the middle of the rumen and into a plastic flask. The samples were transferred into a bottle with thermal insulation at 39°C after being filtered through four layers of folded cheesecloth. Part of the preparation of the medium solution (2,000 mL) contains 0.24 mL of micro-mineral solution, 2.44 mL of resazurine, 99.0 mL of reduction solution, 480.0 mL of macro-mineral solution, 480.0 mL of buffer solution, and 950.0 mL of distilled water, respectively. Under constant CO_2_ flushing, rumen fluid was combined with the medium substrate at 1:2 (mL/mL). Substrates in total (concentrate and roughage sources) were weighed into glass bottles (60 mL), then the respective treatments, MMLE was added at 0.00, 0.02, 0.03, and 0.04 g DM. The bottles were capped with rubber stoppers and aluminum caps. Rumen inocula mixture was added (40 mL) to the bottles and incubated at 39°C, as described in Matra et al [27].

### *In vitro* incubation

During incubation, the production of gas was recorded at 1, 2, 4, 6, 8, 12, 24, 48, 72, and 96 h (3 bottles/treatment). The equation of Ørskov and McDonald [28] was used to analyze all gas production data; Y = a+b (1−e^−ct^), where Y = gas generated at time “t” (mL), a = the gas production from the immediately soluble fraction (mL), b = the gas production from the insoluble fraction (mL), c = the gas production rate constant for the insoluble fraction (mL/h), and t = incubation time (h). The samples were collected separately for pH, microbial population, ammonia nitrogen (NH_3_-N), and VFA analyses at 12, 24, and 48 h-after incubation (2 bottles/treatment). A portable pH meter was used to determine the pH (HANNA Instruments HI 8424 microcomputer, Singapore). The rumen fluid instances were centrifuged at 16,000×g for 15 minutes after being filtered through instances cheesecloth, then to analyze the NH_3_-N concentration using micro-Kjeldahl techniques, the supernatant was kept at −20°C [18] and VFA profiles (HPLC; ETL Testing Laboratory, Inc., Cortland, NY, USA); according to Samuel et al [29]. Additionally, *in vitro* nutrient degradability was measured using a different set (2 bottles/treatment). The production of methane (CH_4_; 3 bottles/treatment) was measured using GC machine (GC-2014; Shimadzu Co Ltd., Kyoto, Japan); methane production (% v/v) = (Peak area/18,108)/0.3, where 0.3 = the volume of gas was kept in the bottle (10 mL) and 18,108 = the slope estimates of the standard methane graph.

### Real-time polymerase chain reaction

Approximately, 1 mL of rumen fluid from *in vitro* study was extracted for total genomic DNA (gDNA) following to the method of QIAamp Fast DNA Stool Mini kit (Qiagen, Hilden, Germany). The gDNA quality (the concentration at ≥50 ng/μL) was indicated by absorbance at OD260/280 = 1.8 to 2.0 using Nanodrop spectrophotometer (Thermo Scientific, USA). The microbial population including *Ruminococcus albus*, *Ruminococcus flavefaciens*, *Fibrobactor succinnogenes*, *Butyribrivio fibrisolvens*, *Megasphaera elsdenii*, and *Methanobacteriales* were identified using the specific primers through real-time polymerase chain reaction (PCR) technique, as shown in Table 1. The real-time PCR amplification and detection were performed by Maxima SYBR Green qPCR Master Mix using Chromo 4TM system (Bio-Rad, Hercules, CA, USA), more detail of the protocols was demonstrated in Koike and Kobayashi [30].

### Statistical analysis

The data were analyzed using the general linear model procedure following to the method of SAS [34], for a CRD; Y_ij_ = μ+τ_i_+ɛ_ij_, where μ = overall mean, τ_i_ = treatment effect, ɛ_ij_ = residual error, and Y_ij_ = observation. The mean values of the experimental treatments were compared with Tukey’s test. Differences between treatment means were reported as statistically different had p-values of <0.05 and <0.01. Trends of MMLE supplementation responses were analyzed by Orthogonal polynomials.

## RESULTS

### Nutritive values and morphological characterization of MMLE

**Figure 1:**
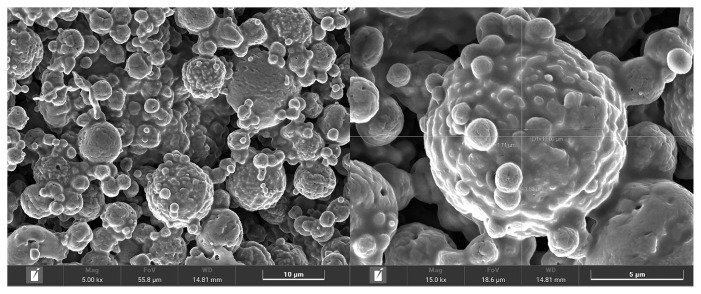
Morphological characterization of microencapsulated-*Mitragyna* leaves extracts.

The nutritive values of MMLE were 90.1% DM, and 96.4%, 18.6%, 72.2%, and 21.9% DM basis for OM, CP, NDF, and ADF, respectively. Importantly, BC contained in MMLE were 307.8 mg gallic acid equivalent/g DM of TPC and 105.3 mg quercetin equivalent/g DM of TFC. In the antioxidant capacity, including 94.8% DPPH, 90.3% ABTS, and 34.4 mg trolox equivalent/g DM of FRAP), as shown in Table 2. Moreover, morphological characterization of MMLE, chitosan microparticles showed that they had entirely spherical surface morphologies, with porous surrounding particle spheres interspersed with smooth and rough surfaces. The MMLE identified numerous particles with sizes ranging from 1.5 to 11.0 μm in diameter (Figure 1).

### *In vitro* gas production kinetics

**Figure 2:**
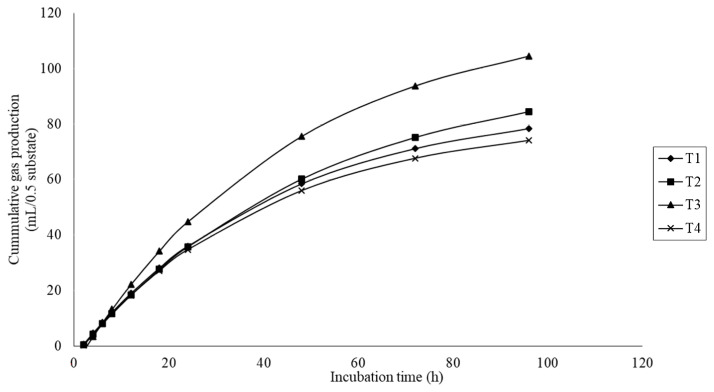
Effect of microencapsulated-*Mitragyna* leaves extracts (MMLE) on cumulative gas production curves after 1–96 h of incubation. The treatments (T1–T4) were added with MMLE at 0%, 4%, 6%, and 8% of total dry matter substrate, respectively.

The gas production results are presented in Table 3. Gas production kinetics, including the gas production from the immediately soluble fraction (a), the potential extent of gas production (a+b), and the gas production from the insoluble fraction (b) were significantly different (quadratic effect; p<0.01) with MMLE supplementation. There was a significant difference (p<0.05) on the gas production rate constant for the insoluble fraction (c), with higher values for the treatment fed 6% MMLE. In addition, the cumulative gas production was quadratically increased (p<0.01) with MMLE addition (Figure 2).

### Nutrient degradability

The MMLE had the greatest *in vitro* dry matter degradability (IVDMD) (p<0.05) at 12, 24, and 48 h of fermentation (quadratic effect) when supplemented with 6% of total DM substrate. The lowest IVDMD occurred with MMLE addition at 8% of total DM substrate. Furthermore, this parameter was not linearly influenced, as presented in Table 3.

### Ruminal pH and NH_3_-N concentration

Table 4, the ruminal pH (12, 24, and 48 h) were not affected (p>0.05), when increasing the level of MMLE. The ammonia nitrogen content (24 and 48 h) was quadratically increased (p<0.05 and p<0.01) when MMLE was added at 6% of total DM substrate. This concentration at 24 and 48 h was significantly higher (p<0.05 and p<0.01) than at 12 h by the supplementation of MMLE.

### Volatile fatty acids and methane production

The 6% MMLE had significantly (quadratic effect; p<0.05) greater acetate, propionate, acetate to propionate ratio, and total VFA production, and it had the highest propionate content (p<0.05) when compared with the control treatments. The content of butyrate did not differ (p>0.05) with MMLE addition. Moreover, methane production (after 12, 24, and 48 h of fermentation) was linearly decreased (p<0.05) when MMLE level was increased. The supplementation of 6% MMLE had the lowest methane content (p<0.05) compared with other treatments, as shown in Table 5.

### Microbial dynamics

Based on species level, MMLE supplementation was able to change the bacterial and archaeal population. The present findings showed that MMLE supplementation increased cellulolytic bacteria, namely *Ruminococcus albus* (p<0.05), *Ruminococcus flavefaciens* (p<0.05), and *Fibrobactor succinnogenes* (p<0.05). The relative abundance of *Butyribrivio fibrisolvens* increased (p<0.05) in 6% MMLE compared with control, while *Megasphaera elsdenii* was not statistically different (p>0.05) among treatments. Importantly, *Methanobacteriales* was linearly decreased (p<0.05) when fed the MMLE (Table 6).

## DISCUSSION

### *In vitro* gas production kinetics

In the present study, gas kinetics, especially the gas production rate constant for the insoluble fraction (c), were improved by the MMLE supplementation. It could be due to the capability of BC to enhance microbial growth and activity and its ability to bind in the contents of protein and fiber [3,35]. Accordingly, Phesatcha et al [10] stated that *Mitragyna* leaves powder enhanced gas production kinetics, it’s possible that it improved the rumen microbe and increased the substrate’s capacity to degrade, so improving the kinetics of gas production.

### Nutrient degradability

The MMLE supplementation to the diet clearly increased IVDMD, which was significantly higher with the addition of 6% of total DM substrate. This might be explained by an increase in the number of microbes, which would cause more feed to breakdown, which was one important role of the BC contained in MMLE. Zhan et al [36] explained that flavonoids and phenolics have a range of biological effects that can impact ruminal microbes, which in turn increases how feed is degraded in the rumen. Sommai et al [37] reported that flavonoid extracts from Alternanthera sissoo supplementation significantly increased *in vitro* degradability.

### Ruminal pH and NH_3_-N concentration

This research has shown that ruminal pH did not influence the treatments. For typical rumen fermentation, microbial growth, and microbial activity, the data were in the normal range (6.85 to 6.99). Accordingly, Wanapat [38] reported that pH ranges between 6.5 and 7.0 are optimum for microbial activity and growth. Strategic addition of phenolic-containing feedstuffs can enhance rumen fermentation by preserving a higher pH [39]. Furthermore, MMLE supplementation was improved NH_3_-N concentration both 12, 24, and 48 h. This may be a result of the plant-bioactive extract’s potential to enhance the proteolysis process. Plant-based bioactive supplementation increases the concentration of ruminal NH_3_-N, which was confirmed by Ahmed et al [40]. Furthermore, it could be a positive effect of the concentrate and MMLE containing protein source at 14.6% and 18.6% CP, thus increasing the amount of NH_3_-N present as a result.

### Volatile fatty acids and methane production

Under this investigation, MMLE supplementation increased the molarity of VFAs especially propionate and total VFA production, while decreased acetate production. Patra and Saxena [41] explained that BC may also cause a change in propionate produced when there is an excess of hydrogen. Hydrogen is used to create propionate instead of being the major substrate for the methane production pathway [42]. These findings agree with Totakul et al [43] who revealed that the *Cnidoscolus* leaves pellet supplementation significantly increased propionate concentration, while decreased acetate to propionate ratio. Propionate content typically increases when rumen methanogenesis is inhibited, and this was also shown in the current investigation. Bodas et al [44] demonstrated that phenolic acids and polyphenols suppress methanogenesis, while also improving fermentation parameters. Therefore, phenolics and flavonoids from multi-functional tropical plants have the potential to directly inhibit methanogen population and activity. Furthermore, BC in feeds has been demonstrated, whether in natural form or as plant extracts, to have an impact on the rumen’s ability to reduce methane production by rumen microorganisms. Cellulolytic bacteria are among the specific microorganisms that BC directly affects. It causes *F. succinogenes* (the non-hydrogen producing bacteria) to produce more propionate and reduce the acetate to propionate ratio [45]. In this study, MMLE supplementation clearly decreased methane production. As described in Chanjula et al [11], dried *Mitragyna* leaves linearly decreased methane production and F. succinogenes quadratically increased when the level of *Mitragyna* leaves was added. Huang et al [46] showed that Paulownia hybrid leaves decreased methane production, it could be the result of a decline in Archaea especially methanogens due to secondary metabolite activities.

### Microbial dynamics

In the current study, the cellulolytic bacteria population increased with the levels of MMLE supplementation. Consequently, the phenolic and flavonoid containing in MMLE, compounds could influence the cellulolytic bacteria activities especially when MMLE was supplemented at 8% of total DM substrate. BC activity alters protein translocation, phosphorylation processes, ion gradients, electron transport, and other enzyme-dependent processes, which results in the impacted cellulolytic bacteria losing chemiosmotic control [47]. Nevertheless, BC should be supplemented at a suitable level for microbe activity, especially cellulolytic bacteria. *F. succinogenes*, *R. albus*, and *R. flavefaciens* have been identified as the main cellulolytic bacterial species in the rumen and more these groups could improve ruminant degradation of fiber [48]. According to Chanjula et al [11] stated that dried *Mitragyna* leaves was enhanced *F. succinogenes*, *R. albus*, and *R. flavefaciens*. Huang et al [46] revealed that Paulownia hybrid leaves containing flavonoid and phenolic compounds increases total bacteria, as well as in particular species of *B. fibrisolvens* and *F. succinogenes*. This may be explained by the ruminal microbes’ response to the flavonoids and phenolics, perhaps as a result of hydrogenation, which transforms toxic compounds into less toxic forms [49]. Moreover, MMLE addition increased *Butyrivibrio fibrisolvens* group, while reduced methanogens group (*Methanobacteriales*), which could be attributed to the availability of BC in the MMLE. Similarly, Phesatcha et al [10] showed that the supplementation of *Mitragyna* leaves reduced ruminal methanogens population and methane production. BC has an immediate impact on rumen methanogens, by interacting with the proteinaceous adhesin, suppressing methanogen growth, reducing interspecies hydrogen transfer, and inhibiting the methanogen-protozoa complex’s formation [50].

## CONCLUSION

Based on the findings, supplementation of MMLE at 6% of total DM substrate enhanced rumen nutrient degradability, fermentation end-products especially propionate production, and decreased methanogens and methane production. Hence, MMLE could be an effective dietary BC and could have the potential to be used for ruminant feed additives.

## Figures and Tables

**Table 1:** The specific primers of rumen microbes

Species	Specific primers	Primer sequences (5′-3′)	PCR products (bp)	References
*Fibrobacter succinogenes*	Fs219f	GGTATGGGATGAGCTTGC	446	Koike and Kobayashi [30]
	Fs654r	GCCTGCCCCTGAACTATC		
*Ruminococcus albus*	Ra1281f	CCCTAAAAGCAGTCTTAGTTCG	175	
	Ra1439r	CCTCCTTGCGGTTAGAACA		
*Ruminococcus flavefaciens*	Rf154f	TCTGGAAACGGATGG TA	295	
	Rf425r	CCTTTAAGACAGGAGTTTACA A		
*Megasphaera elsdenii*	Mef	GACCGAAACTGCGATGCTAGA	128	Ouwerkerk et al [31]
	Mer	TCCAGAAAGCCGCTTTCGCCACT		
*Butyrivibrio fibrisolvens*	Bff	CGCATGATGCAGTGTGAAAAGCTC	625	Fernando et al [32]
	Bfr	CCTCCCGACACCTATTATTCATCG		
*Methanobacteriales*	Mbt857f	GGGCTTGCTTTGGAAACTGTT	343	Yu et al [33]
	Mbt1196r	CCCACCGATGTTCCTCCTAA		

PCR, polymerase chain reaction; bp, base pair.

**Table 2:** Chemical composition of feed used in the experiment

Items	Concentrate	Rice straw	MLM	MMLE
Ingredients (% as fed)				
Cassava chip	54.0			
Rice bran meal	17.0			
Palm kernel meal	13.0			
Soybean meal	10.5			
Urea	2.5			
Sulphur	1.0			
Salt	1.0			
Mineral mixed^1)^	1.0			
Chemical composition				
DM (%)	90.5	89.4	93.1	90.1
	-------------------------------------------------------------------- % DM --------------------------------------------------------------------			
OM	92.2	85.4	94.8	96.4
Crude protein	14.6	2.4	19.7	18.6
Neutral-detergent fiber	20.5	78.9	48.0	72.2
Acid-detergent fiber	8.2	52.6	19.6	21.9
Phytonutrient compound				
TPC (mg GAE/g DM)	-	-	306.9	307.8
TFC (mg QUE/g DM)	-	-	119.2	105.3
Antioxidant capacity				
DPPH (%)	-	-	91.4	94.8
ABTS (%)	-	-	95.3	90.3
FRAP (mg TROE/g DM)	-	-	39.0	34.4

MLM, *Mitragyna* leaves meal; MMLE, microencapsulated-*Mitragyna* leaves extracts; DM, dry matter; OM, organic matter; TPC, total phenolic content; TFC, total flavonoid content; GAE, gallic acid equivalent; QUE, quercetin equivalent; DPPH, 2, 2-diphenyl-1-picrylhydrazyl as DPPH radical scavenging activity; ABTS, 2, 2′-azino-bis (3-ethylbenzothiazoline-6-sulfonic acid) as ABTS radical scavenging activity; FRAP, ferric reducing antioxidant power; TROE, trolox equivalent.

1) Mineral premix (contains per kg): vitamin A 10,000,000 IU; vitamin D 1,600,000 IU; vitamin E 70,000 IU; Fe 50 g; Mn 40 g; Zn 40 g; Cu 10 g; I 0.5 g; Se 0.1 g; Co 0.1 g.

**Table 3:** Supplementation of microencapsulated-Mitragyna leaves extracts on gas kinetics and nutrient degradability

MMLE (% of total substrate)	Gas kinetics^1)^				Cumulative gas^2)^ at 96 h	IVDMD (% DM)		
	
	a	b	c	a+b		12 h	24 h	48 h
0	−4.1^a^	85.4^a^	0.025^a^	81.3^a^	81.2^a^	57.9^a^	62.8^a^	68.8^a^
4	−3.6^b^	91.1^b^	0.027^b^	87.6^b^	87.5^b^	64.1^b^	68.9^b^	73.6^b^
6	−3.6^b^	105.5^c^	0.034^c^	101.9^c^	101.6^c^	64.5^b^	69.1^b^	73.9^b^
8	−3.9^b^	81.8^d^	0.022^d^	77.9^d^	77.8^d^	60.2^c^	63.1^c^	70.8^c^
SEM	0.32	1.43	0.02	1.56	1.73	0.85	0.87	0.91
Orthogonal polynomials								
Linear	0.13	0.05	0.11	0.13	0.09	0.17	0.19	0.60
Quadratic	<0.01	<0.01	0.02	<0.01	<0.01	0.03	0.04	0.04
Cubic	0.25	0.73	0.06	0.86	0.57	0.16	0.17	0.86

MMLE, microencapsulated-*Mitragyna* leaves extracts; IVDMD, *in vitro* dry matter degradability; SEM, standard error of mean.

1) Gas production kinetics, a, the gas production from the immediately soluble fraction (mL); b, the gas production from the insoluble fraction (mL); c, the gas production rate constant for the insoluble fraction (mL/h); a+b, the potential extent of gas production (mL).

2) Cumulative gases at 96 h (mL/0.2 g DM substrate).

a–d Means within the same column with different letters are significantly different at p<0.05.

**Table 4:** Supplementation of microencapsulated-*Mitragyna* leaves extracts on ruminal pH and ammonia-nitrogen concentration

MMLE (% of total substrate)	pH			Ammonia nitrogen (mg/dL)		
	
	12 h	24 h	48 h	12 h	24 h	48 h
0	6.96	6.90	6.89	9.7^a^	10.5^a^	13.1^a^
4	6.94	6.94	6.92	12.0^b^	11.7^b^	14.9^b^
6	6.96	6.95	6.91	12.6^b^	12.8^c^	16.8^c^
8	6.99	6.95	6.94	8.7^a^	9.5^a^	13.3^a^
SEM	0.01	0.01	0.02	0.25	0.23	0.46
Orthogonal polynomials						
Linear	0.28	0.14	0.05	0.41	0.61	0.83
Quadratic	0.34	0.43	0.10	<0.01	0.02	<0.01
Cubic	0.65	0.95	0.17	0.21	0.05	0.58

MMLE, microencapsulated-*Mitragyna* leaves extracts; SEM, standard error of mean.

a–c Means within the same column with different letters are significantly different at p<0.05.

**Table 5:** Supplementation of microencapsulated-*Mitragyna* leaves extracts on volatile fatty acids and methane production

MMLE (% of total substrate)	VFA (mol/100 mL)			C_2_:C_3_	Total VFA (mmol/L)	Methane production (%)		
	
	C_2_	C_3_	C_4_			12 h	24 h	48 h
0	69.4^a^	23.7^a^	6.9	2.95^a^	67.4^a^	27.5^a^	30.8^a^	34.9^a^
4	68.5^b^	24.9^b^	6.6	2.75^b^	74.1^b^	26.6^b^	29.9^b^	34.0^b^
6	65.0^c^	26.9^c^	8.1	2.40^c^	84.8^c^	25.1^c^	28.4^c^	33.5^c^
8	69.2^a^	24.5^b^	6.3	2.85^a^	71.7^b^	24.9^d^	27.6^d^	32.3^d^
SEM	0.46	0.37	0.25	0.08	2.25	0.07	0.05	0.05
Orthogonal polynomials								
Linear	0.42	0.53	0.88	0.50	0.37	0.02	0.01	0.01
Quadratic	0.04	0.03	0.21	0.04	0.04	0.62	0.48	0.51
Cubic	0.08	0.16	0.10	0.34	0.64	0.58	0.35	0.45

MMLE, microencapsulated-*Mitragyna* leaves extracts; VFA, volatile fatty acids; C_2_, acetate; C_3_, propionate; C_4_, butyrate; C_2_:C_3_, acetate to propionate ratio; SEM, standard error of mean.

a–d Means within the same column with different letters are significantly different at p<0.05.

**Table 6:** Supplementation of microencapsulated-*Mitragyna* leaves extracts on rumen microbial population

Species	Incubation time (h)	MMLE (% of total substrate)				SEM	Orthogonal polynomials		
	
		0	4	6	8		L	Q	C
*Fibrobacter succinogenes* (Log copies/mL)	12	5.3^a^	5.7^b^	5.9^c^	5.6^b^	0.38	0.11	0.04	0.24
	24	5.5^a^	6.1^b^	6.3^c^	6.0^b^	0.34	0.24	0.02	0.97
	48	5.7^a^	6.2^b^	6.5^c^	6.0^b^	0.26	0.62	0.01	0.69
*Ruminococcus albus* (Log copies/mL)	12	7.3^a^	7.7^b^	8.2^c^	7.5^b^	0.55	0.21	0.03	0.69
	24	7.7^a^	7.9^a^	8.5^b^	7.8^a^	0.46	0.17	0.02	0.87
	48	7.9^a^	8.1^a^	8.8^b^	8.0^a^	0.62	0.52	0.04	0.13
*Ruminococcus flavefaciens* (Log copies/mL)	12	6.6^a^	6.8^a^	7.3^b^	6.7^a^	0.53	0.57	0.03	0.57
	24	6.8^a^	6.9^a^	7.6^b^	6.8^a^	0.44	0.24	0.03	0.94
	48	6.8^a^	7.0^a^	7.9^b^	6.9^a^	0.35	0.15	0.01	0.32
*Megasphaera elsdenii* (Log copies/mL)	12	7.1	7.0	7.0	7.0	1.19	0.78	0.62	0.69
	24	7.1	7.1	7.2	7.1	1.25	0.56	0.45	0.83
	48	7.0	6.8	6.8	6.8	0.88	0.11	0.51	0.49
*Butyrivibrio fibrisolvens* (Log copies/mL)	12	6.2	6.1	6.0	6.0	1.52	0.37	0.35	0.91
	24	6.2	6.4	6.6	6.5	0.46	0.29	0.46	0.80
	48	6.2^a^	6.3^a^	6.8^b^	6.4^a^	0.34	0.28	0.01	0.88
*Methanobacteriales* (Log copies/mL)	12	7.1^a^	6.9^b^	6.5^c^	6.5^c^	0.37	0.02	0.40	0.94
	24	7.2^a^	6.8^a^	6.4^b^	6.3^b^	0.56	0.03	0.70	0.45
	48	7.4^a^	6.6^b^	5.9^c^	5.8^c^	0.48	0.03	0.50	0.16

MMLE, microencapsulated-*Mitragyna* leaves extracts; SEM, standard error of mean; L, linear; Q, quadratic; C, cubic.

a–c Means within the same row with different letters are significantly different at p<0.05.

## References

[1] Kholif AE, Gouda GA, Abu Elella AA, Patra AK (2022). Replacing the concentrate feed mixture with Moringa oleifera leaves silage and Chlorella vulgaris microalgae mixture in diets of damascus goats: lactation performance, nutrient utilization, and ruminal fermentation. Animal.

[2] Matra M, Wanapat M (2022). Phytonutrient pellet supplementation enhanced rumen fermentation efficiency and milk production of lactating Holstein-Friesian crossbred cows. Anim Nutr.

[3] Wanapat M, Viennasay B, Matra M (2021). Supplementation of fruit peel pellet containing phytonutrients to manipulate rumen pH, fermentation efficiency, nutrient digestibility and microbial protein synthesis. J Sci Food Agric.

[4] Singh S, Hundal JS, Patra AK, Sethi RS, Sharma A (2022). A composite polyphenol-rich extract improved growth performance, ruminal fermentation and immunity, while decreasing methanogenesis and excretion of nitrogen and phosphorus in growing buffaloes. Environ Sci Pollut Res.

[5] Vasta V, Luciano G (2011). The effects of dietary consumption of plants secondary compounds on small ruminants’ products quality. Small Rumin Res.

[6] Eisenman SW, Raffa RB (2015). The botany of Mitragyna speciosa (Korth.) Havil. and related species. Kratom and other Mitragynines: The chemistry and pharmacology of opioids from a non-opium source.

[7] Singh D, Chear NJY, Narayanan S (2020). Patterns and reasons for kratom (Mitragyna speciosa) use among current and former opioid poly-drug users. J Ethnopharmacol.

[8] Raffa RB (2015). Kratom and other mitragynines: the chemistry and pharmacology of opioids from a non-opium source.

[9] Hassan Z, Muzaimi M, Navaratnam V (2013). From Kratom to mitragynine and its derivatives: Physiological and behavioural effects related to use, abuse, and addiction. Neurosci Biobehav Rev.

[10] Phesatcha K, Phesatcha B, Wanapat M, Cherdthong A (2022). Mitragyna speciosa korth leaves supplementation on feed utilization, rumen fermentation efficiency, microbial population, and methane production in vitro. Fermentation.

[11] Chanjula P, Wungsintaweekul J, Chiarawipa R (2022). Effect of feed supplement containing dried kratom leaves on apparent digestibility, rumen fermentation, serum antioxidants, hematology, and nitrogen balance in goats. Fermentation.

[12] Kim TB, Lee JS, Cho SY, Lee HG (2020). In vitro and in vivo studies of rumen-protected microencapsulated supplement comprising linseed oil, vitamin e, rosemary extract, and hydrogenated palm oil on rumen fermentation, physiological profile, milk yield, and milk composition in dairy cows. Animal.

[13] Vakarelova M, Zanoni F, Lardo P (2017). Production of stable food-grade microencapsulated astaxanthin by vibrating nozzle technology. Food Chem.

[14] Flores FP, Singh RK, Kerr WL, Pegg RB, Kong F (2014). Total phenolics content and antioxidant capacities of microencapsulated blueberry anthocyanins during in vitro digestion. Food Chem.

[15] Nouri A (2019). Chitosan nano-encapsulation improves the effects of mint, thyme, and cinnamon essential oils in broiler chickens. Br Poult Sci.

[16] Kurek MA, Pratap-Singh A (2020). Plant-based (hemp, pea and rice) protein–maltodextrin combinations as wall material for spray-drying microencapsulation of hempseed (Cannabis sativa) oil. Foods.

[17] Ko JA, Park HJ, Hwang SJ, Park JB, Lee JS (2002). Preparation and characterization of chitosan microparticles intended for controlled drug delivery. Int J Pharm.

[18] AOAC (2012). Official methods of analysis.

[19] Van Soest PV, Robertson JB, Lewis BA (1991). Methods for dietary fiber, neutral detergent fiber, and nonstarch polysaccharides in relation to animal nutrition. J Dairy Sci.

[20] Al-Duais M, Müller L, Böhm V, Jetschke G (2009). Antioxidant capacity and total phenolics of Cyphostemma digitatum before and after processing: use of different assays. Eur Food Res Technol.

[21] Topçu G, Ay M, Bilici A, Sarıkürkcü C, Öztürk M, Ulubelen A (2007). A new flavone from antioxidant extracts of Pistacia terebinthus. Food Chem.

[22] Gali L, Bedjou F (2019). Antioxidant and anticholinesterase effects of the ethanol extract, ethanol extract fractions and total alkaloids from the cultivated Ruta chalepensis. S Afr J Bot.

[23] Re R, Pellegrini N, Proteggente A, Pannala A, Yang M, Rice-Evans C (1999). Antioxidant activity applying an improved ABTS radical cation decolorization assay. Free Radic Biol Med.

[24] Benzie IF, Strain JJ (1996). The ferric reducing ability of plasma (FRAP) as a measure of “antioxidant power”: the FRAP assay. Anal Biochem.

[25] Phupaboon S, Matra M, Prommachart R, Totakul P, Supapong C, Wanapat M (2022). Extraction, characterization, and chitosan microencapsulation of bioactive compounds from Cannabis sativa L., Cannabis indica L., and Mitragyna speiosa K. Antioxidants.

[26] National Research Council (NRC) (2001). Nutrient requirements of dairy cattle.

[27] Matra M, Totakul P, Wanapat M (2021). Utilization of dragon fruit waste by-products and non-protein nitrogen source: Effects on in vitro rumen fermentation, nutrients degradability and methane production. Livest Sci.

[28] Ørskov ER, McDonald I (1979). The estimation of protein degradability in the rumen from incubation measurements weighted according to rate of passage. J Agric Sci.

[29] Samuel M, Ceballos-Baumann AO, Blin J (1997). Evidence for lateral premotor and parietal overactivity in Parkinson’s disease during sequential and bimanual movements. A PET study. Brain.

[30] Koike S, Kobayashi Y (2001). Development and use of competitive PCR assays for the rumen cellulolytic bacteria: Fibrobacter succinogenes, Ruminococcus albus and Ruminococcus flavefaciens. FEMS Microbiol Lett.

[31] Ouwerkerk D, Klieve AV, Forster RJ (2002). Enumeration of Megasphaera elsdenii in rumen contents by real-time Taq nuclease assay. J Appl Microbiol.

[32] Fernando SC, Purvis HT, Najar FZ (2010). Rumen microbial population dynamics during adaptation to a high-grain diet. Appl Environ Microbiol.

[33] Yu Y, Lee C, Kim J, Hwang S (2005). Group-specific primer and probe sets to detect methanogenic communities using quantitative real-time polymerase chain reaction. Biotechnol Bioeng.

[34] Statistical Analysis System (2013). User’s guide: Statistic.

[35] Pal K, Patra AK, Sahoo A (2015). Evaluation of feeds from tropical origin for in vitro methane production potential and rumen fermentation in vitro. Span J Agric Res.

[36] Zhan J, Liu M, Su X, Zhan K, Zhang C, Zhao G (2017). Effects of alfalfa flavonoids on the production performance, immune system, and ruminal fermentation of dairy cows. Asian-Australas J Anim Sci.

[37] Sommai S, Cherdthong A, Suntara C, So S, Wanapat M, Polyorach S (2021). In vitro fermentation characteristics and methane mitigation responded to flavonoid extract levels from Alternanthera sissoo and dietary ratios. Fermentation.

[38] Wanapat M (2003). Manipulation of cassava cultivation and utilization to improve protein to energy biomass for livestock feeding in the tropics. Asian-Australas J Anim Sci.

[39] Viennasay B, Totakul P, Matra M, Phesatcha B, Wanapat M (2022). Influence of bamboo grass (Tiliacora triandra, Diels) pellet supplementation on in vitro fermentation and methane mitigation. J Sci Food Agric.

[40] Ahmed E, Fukuma N, Hanada M, Nishida T (2021). The efficacy of plant-based bioactives supplementation to different proportion of concentrate diets on methane production and rumen fermentation characteristics in vitro. Animals.

[41] Patra AK, Saxena J (2009). The effect and mode of action of saponins on the microbial populations and fermentation in the rumen and ruminant production. Nutr Res Rev.

[42] Newbold CJ, López S, Nelson N, Ouda JO, Wallace RJ, Moss AR (2005). Propionate precursors and other metabolic intermediates as possible alternative electron acceptors to methanogenesis in ruminal fermentation in vitro. Br J Nutr.

[43] Totakul P, Viennasay B, Sommai S, Matra M, Infascelli F, Wanapat M (2022). Chaya (Cnidoscolus aconitifolius, Mill. Johnston) pellet supplementation improved rumen fermentation, milk yield and milk composition of lactating dairy cows. Livest Sci.

[44] Bodas R, Prieto N, García-González R, Andrés S, Giráldez FJ, López S (2012). Manipulation of rumen fermentation and methane production with plant secondary metabolites. Anim Feed Sci Technol.

[45] Naumann HD, Tedeschi LO, Zeller WE, Huntley NF (2017). The role of condensed tannins in ruminant animal production: advances, limitations and future directions. Rev Bras Zootec.

[46] Huang H, Szumacher-Strabel M, Patra AK (2021). Chemical and phytochemical composition, in vitro ruminal fermentation, methane production, and nutrient degradability of fresh and ensiled Paulownia hybrid leaves. Anim Feed Sci Technol.

[47] Ultee A, Kets EPW, Smid EJ (1999). Mechanisms of action of carvacrol on the food-borne pathogen Bacillus cereus. Appl Environ Microbiol.

[48] Rira M, Morgavi DP, Archimède H (2015). Potential of tannin-rich plants for modulating ruminal microbes and ruminal fermentation in sheep. J Anim Sci.

[49] Berchez M, Urcan AC, Corcionivoschi N, Criste A (2019). In vitro effects of phenolic acids and IgY immunoglobulins on aspects of rumen fermentation. Rom Biotechnol Lett.

[50] Manasri N, Wanapat M, Navanukraw C (2012). Improving rumen fermentation and feed digestibility in cattle by mangosteen peel and garlic pellet supplementation. Livest Sci.

